# Semantically-Enabled Sensor Plug & Play for the Sensor Web

**DOI:** 10.3390/s110807568

**Published:** 2011-08-02

**Authors:** Arne Bröring, Patrick Maúe, Krzysztof Janowicz, Daniel Nüst, Christian Malewski

**Affiliations:** 1 Institute for Geoinformatics, University of Münster, D-48151 Münster, Germany; E-Mails: pajoma@uni-muenster.de (P.M.); daniel.nuest@uni-muenster.de (D.N.); c.malewski@uni-muenster.de (C.M.); 2 ITC Faculty, University of Twente, 7522 NB Enschede, The Netherlands; 3 52° North Initiative for Geospatial Open Source Software, D-48155 Münster, Germany; 4 Geography Department, University of California, Santa Barbara, CA 93106, USA; E-Mail: jano@geog.ucsb.edu

**Keywords:** Sensor Web Enablement, Sensor Plug & Play, sensor integration, Semantic Web, sensor bus, sensor interface descriptors, semantic matching, ontology alignment

## Abstract

Environmental sensors have continuously improved by becoming smaller, cheaper, and more intelligent over the past years. As consequence of these technological advancements, sensors are increasingly deployed to monitor our environment. The large variety of available sensor types with often incompatible protocols complicates the integration of sensors into observing systems. The standardized Web service interfaces and data encodings defined within OGC’s Sensor Web Enablement (SWE) framework make sensors available over the Web and hide the heterogeneous sensor protocols from applications. So far, the SWE framework does not describe how to integrate sensors on-the-fly with minimal human intervention. The driver software which enables access to sensors has to be implemented and the measured sensor data has to be manually mapped to the SWE models. In this article we introduce a *Sensor Plug & Play* infrastructure for the Sensor Web by combining (1) semantic matchmaking functionality, (2) a publish/subscribe mechanism underlying the SensorWeb, as well as (3) a model for the declarative description of sensor interfaces which serves as a generic driver mechanism. We implement and evaluate our approach by applying it to an oil spill scenario. The matchmaking is realized using existing ontologies and reasoning engines and provides a strong case for the semantic integration capabilities provided by Semantic Web research.

## 1. Introduction

The Sensor Web envisions uniform access to sensor resources comparable to the retrieval of information resources in the World Wide Web today [1]. It will eventually enable Web-based discovery, access, exchange, and processing of sensor observations, as well as the tasking of sensor systems. The Sensor Web Enablement (SWE) initiative of the Open Geospatial Consortium (OGC) defines standards to build such a Sensor Web [2]. SWE makes sensors available over the Web through well-defined formats and Web service interfaces by hiding the sensor communication details and the heterogeneous sensor protocols from applications working on top of these services. Deploying a SWE framework is especially appropriate for large information infrastructures where multiple participating organizations (or multiple departments of the same organization) are required to work with a shared set of sensor resources. In the recent years, SWE standards have been applied in various research projects and systems showing their practicability and suitability in real world applications. Such applications range from a debris flow monitoring system for Taiwan [3], the utilization of SWE in disaster management systems [4–6], to a Tsunami early warning system for Indonesia [7]. Nevertheless, substantial effort is required to make a sensor and its observations available on the Sensor Web, since methods and mechanisms to automate this process are missing.

Connecting sensors to the Sensor Web involves three major steps: (i) a sensor description has to be manually registered at a Sensor Web service; (ii) to upload sensor data, driver software needs to be implemented which converts measured data from the native sensor protocol to higher level Sensor Web observations; (iii) sensor characteristics and the service model have to match before sensor observations can be uploaded to a Sensor Web service. For example, the sensor output and the observed property of a feature residing on the Sensor Web have to match. So far, these matchings have to be established and maintained manually by the service provider.

Minimizing adaptation and administration efforts is a major pre-requisite for the on-the-fly integration of sensor data—named *Sensor Plug & Play* here. Based on such a plug & play paradigm, as envisioned in our previous work [8] and also by Pathan *et al*. [9], Sensor Web services can be set up for certain geographic regions and thematic topics. The existence of a new sensor on the Sensor Web triggers an automatic registration with SWE services interested in the sensor’s characteristics. Finally, the sensor can upload its observations which get automatically mapped to the appropriate service model. The automatic registration and mediation is a key to disaster management where an *ad hoc* densification of a sensor network is required. Use cases range from flooding scenarios, in which the affected river courses are not densely enough covered with water gages, to incidents in nuclear plants where *ad hoc* deployments of radiation detectors might be necessary. If Sensor Web services are in place and used by disaster relief organizations as a coherent infrastructure to access observation data, the efficient integration of new sensors becomes crucial. Other areas, where plug & play integration of sensors with existing information infrastructures would facilitate workflows, range from environmental monitoring over early warning systems to precision agriculture.

This research combines previous results with a framework for semantically-enabled matchmaking and mediation based on Semantic Web technologies to realize Sensor Plug & Play. First, we extend the Sensor Bus [10], an intermediary layer between sensors and SWE services, to introduce a publish/subscribe mechanism within the Sensor Web. This is required to make services aware of new sensors appearing on the Sensor Web. Second, a *driver* mechanism for sensors is incorporated in our approach—the Sensor Interface Descriptor (SID) concept [11]. The SID model extends OGC’s SensorML standard to describe the protocol of a particular sensor type in a declarative way. By means of a generic SID interpreter, the native sensor protocol can be translated to the SWE protocols. Third, we analyze the central challenge for Sensor Plug & Play [8], the matchmaking between sensor characteristics and requirements of Sensor Web services, and the automatic inclusion of sensor data into domain specific service models. For example, a sensor measuring precipitation needs to be automatically linked to a service that is interested in weather related observations for incorporation into forecasting models. We tackle this challenge by building a semantically-enabled matchmaking and mediation framework for the Sensor Web. Besides setting the theoretical ground for our work, we provide a working implementation for all components and demonstrate their interaction. To our best knowledge, this is the first working Sensor Plug & Play infrastructure.

To demonstrate our approach we apply the developed methods to a case study. We assume that a release of crude oil from an offshore platform in the Norwegian Sea has caused a spill endangering the marine biosphere. Multiple organizations contribute to the disaster management process. Sensors are deployed to sample weather related properties such as wind direction, wind speed, and air temperature, as well as sea related properties such as water temperature, current direction, current speed, and salinity. Those environmental properties can serve as input parameters to multi-dimensional oil weathering models [12] to simulate the dispersion, spreading, advection, evaporation, emulsification, and dissolution of the oil slick. This information is important to react with appropriate spill response actions (see e.g., [13,14]).

**Figure 1.**
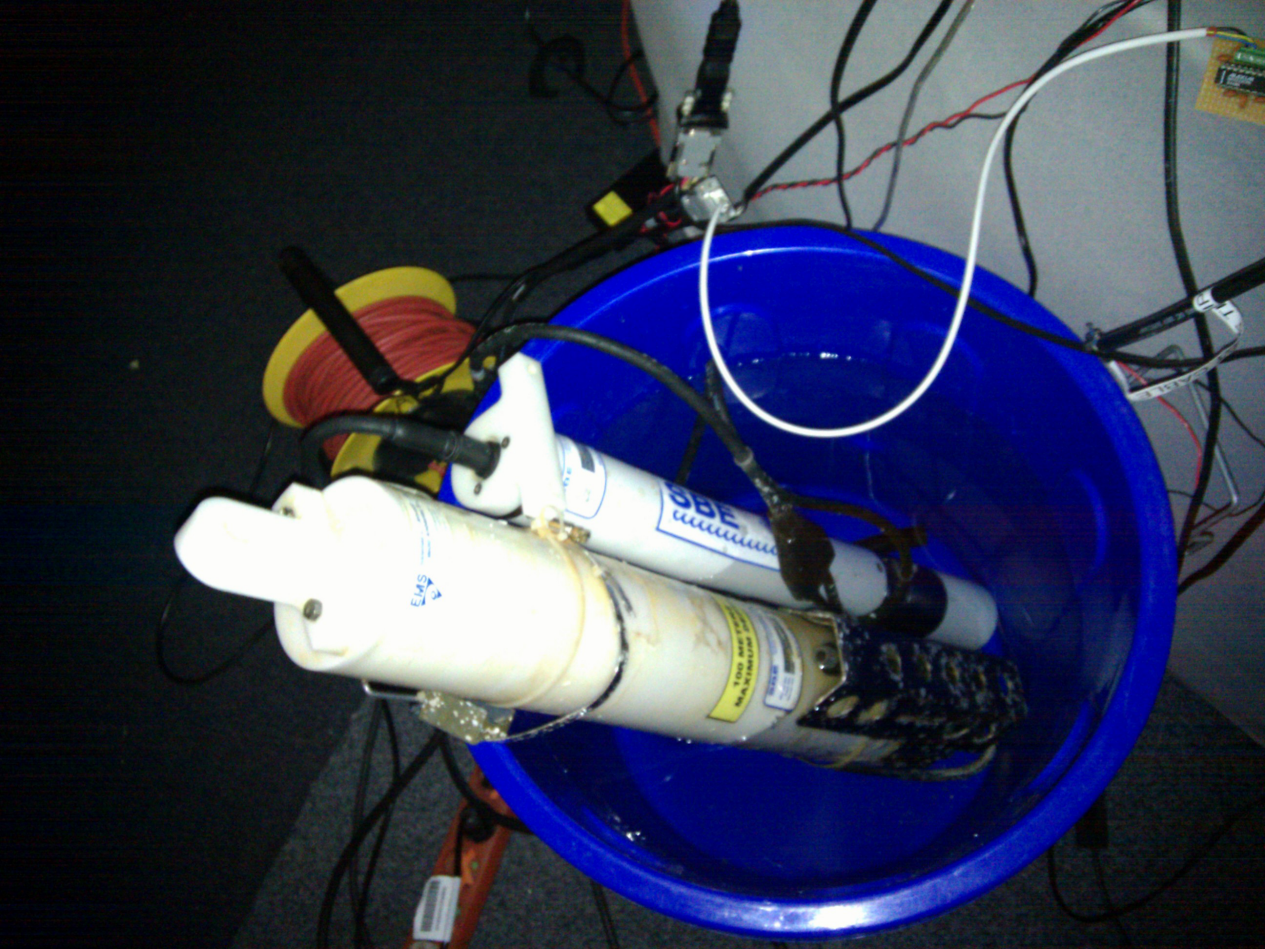
The CTD sensors used in the case study.

We assume that a Sensor Web infrastructure is already in place for the oil spill scenario and used by different disaster management organizations. Newly deployed sensors have to be made available on-the-fly to ensure that applications can directly utilize the gathered observations (e.g., to incorporate them into simulations). As a proof-of-concept, we use typical marine conductivity-temperature-depth (CTD) sensors [15], an *RBR XR-420* and a *Seabird SBE 37*; see Figure 1. Such CTD sensors are commonly used in marine research and can be carried by ships in oil spill scenarios for assessing salinity and temperature at various sampling points of the affected area. The Sensor Web infrastructure is set up based on the developed mechanisms for Sensor Plug & Play and utilized to automatically connect the CTD sensors with Sensor Web services.

The remaining paper is structured as follows. Section 2 introduces the Sensor Web Enablement initiative as well as Semantic Web technologies and ontologies. Section 3 provides an in-depth analysis of requirements for a Sensor Plug & Play on the Sensor Web. Next, Section 4 presents the designed architecture which fulfills the requirements and is followed by a description of the implementation (Section 5). In Section 6, we apply the developed methods to the oil spill scenario and demonstrate their usability by automatically plugging two CTD sensors into a local Sensor Web infrastructure. The paper closes with conclusions and an outlook to future work.

## 2. Background

This section introduces research on Sensor Web Enablement and the Semantic Web relevant for the understanding of the presented work.

### 2.1. Sensor Web Enablement

The Sensor Web Enablement (SWE) initiative of the Open Geospatial Consortium (OGC) has established a suite of standards to realize the vision of a Sensor Web. In this context, the *Sensor Web* is understood as a Web service-based infrastructure which enables the discovery of sensor related resources, the access to sensor observations, the tasking of sensors, as well as eventing and alerting within a Sensor Web environment [16]. Those functionalities are provided through standardized Web service interfaces and data encodings defined by SWE. By establishing such a well-defined and interoperable Sensor Web layer, the heterogeneous sensor protocols and communication details of the underlying sensors are hidden from applications and users.

The current design of the SWE framework consists of the following Web services: First, the Sensor Observation Service (SOS) [17,18] enables access to measured sensor data and sensor metadata. While the SOS follows a pull-based communication paradigm, the Sensor Alert Service (SAS) [19] and its successor the Sensor Event Service (SES) [20] push sensor data to subscribed clients and supports user-defined filter criteria. The Sensor Planning Service (SPS) [21] enables tasking of sensors (e.g., setting the sampling rate of a sensor). Discovery of sensor related resources is provided by the Sensor Instance Registry (SIR) [22] and Sensor Observable Registry (SOR) [23].

In this work, we focus on the SOS as it is the most relevant service in practical applications so far. The interface of the SOS supports access to heterogeneous sensor types, stationary as well as mobile sensors, which gather their data *in situ* or remotely. The heterogeneous communication protocols and data formats of the associated sensors are hidden by the standardized interface. To associate the SOS with a sensor, a description of this sensor is uploaded to the SOS via the *RegisterSensor* operation which can be accessed subsequently by calling the *DescribeSensor* operation. The *InsertObservation* operation is responsible for the integration of newly measured data. Those uploaded observations can be requested by calling the *GetObservation* operation using a combination of temporal, spatial, and thematic filters. The Sensor Model Language (SensorML) [24] is used within SWE to describe the sensor’s characteristics. Observed sensor data is modeled and encoded conforming to the Observations & Measurements (O&M) [25] standard.

**Figure 2.**
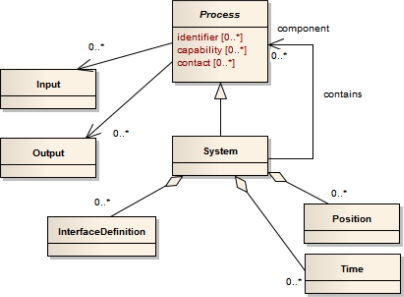
Simplified depiction of an excerpt of the SensorML model.

SensorML specifies a model and XML encoding for the description of sensor related processes. Physical sensors, ranging from simple sensors such as thermometers to composed instruments consisting of multiple single sensing components, as well as virtual sensors can be described. The common root is the abstract type *Process* which converts an input into an output; see Figure 2. Although virtual sensors such as post processing procedures, simulations or environmental models can be in principal described with SensorML, this article focuses on physical sensors. A physical sensor has typically some physical phenomenon (e.g., temperature, conductivity, or pressure) as its input and the quantification of another phenomenon as its output (e.g., water temperature, salinity, or depth). Various metadata about the process can be specified, including its identification, classification, or contact information of the responsible provider. To model physical sensors, the *System* type can be used which also allows to describe it by spatial (e.g., the definition of a geographic position) and temporal (e.g., the definition of a sampling time) attributes. Further, the interfaces of a sensor can be described by means of the *InterfaceDefinition* which allows to describe the Open System Interconnection (OSI) [26] stack of an interface. While this SensorML element provides a means for giving a basic description of a sensor interface, a more detailed interface description including the definition of the protocol structure is not defined by the SensorML model. This is where the Sensor Interface Descriptor (SID) model comes into play to extend SensorML (Section 4.2).

**Figure 3.**
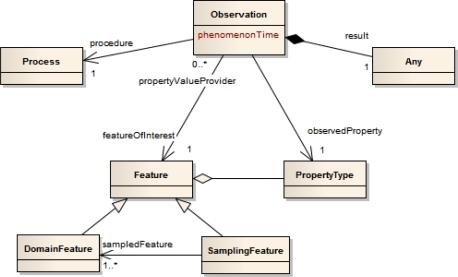
Simplified depiction of O&M’s basic observation model.

O&M defines a model describing observations as an act of observing a certain phenomenon. The basic observation model is shown in Figure 3. An *observation* has a relationship to a *procedure* representing the process which has performed the observation, e.g., often a sensor or instrument, but may also be a human observer or a computation. The *observed property* points to a description of the phenomenon which is observed (e.g., “water temperature” or “salinity”). This phenomenon is a property of the *feature of interest* as the target of an observation. The observation provides a value for this observed property at a certain time, the *phenomenon time*. The observation value is contained in the *result* element. This result can be of any type, ranging from a single measurement to an n-dimensional coverage of values. Subtypes of the basic observation further define this result type.

The feature of interest of an observation can either be represented by a *domain feature* or by a *sampling feature*. A domain feature (e.g., “Norwegian Sea” or “Mississippi River”) is designed for a particular application domain. A sampling feature (e.g., “Station”, “Trajectory”, or “Scene”) is domain independent and purely an artifact of the sampling strategy to produce observations for a domain feature. The sampling feature links to the domain feature which represents the spatially distributed real world entity. For example, when measuring the water temperature of the Norwegian Sea at different depths, the concrete locations of the measurements are represented through three dimensional sampling points. Those sampling points reference the feature representation of the Norwegian Sea which carries the water temperature property. This example shows that the observed property of an observation can either be a direct or transitive member property of the feature of interest. That means, if the feature of interest is a sampling feature, the observed property has to be either a member of the sampling feature or of the domain feature.

### 2.2. Semantic Web Technologies and Ontologies

The Semantic Web describes a stack of methods and technologies to make the meaning of data more explicit. With moving the business logics towards the data, developing applications can be reduced to the combination of existing software components such as Semantic Web reasoners, querying endpoints, or faceted browsing user interfaces. Recently, Vilches-Blazquez *et al*. [27] demonstrated how the interplay of these Semantic Web technologies with annotated geographic information can be used to create semantically-enabled browsers for the Semantic Geospatial Web. Various knowledge representation languages, such as the Web Ontology Language (OWL) or the Resource Description Framework (RDF), are used to make the data available in a machine-readable and understandable way as well as to specify ontologies that restrict the meaning of terms towards their intended representations.

Within the last years, several ontologies have been developed to address the vision of a Semantic Sensor Web [28]. These ontologies range from sensor-centric approaches inspired by SensorML [29], over observation-centric specifications based on O&M [30–32], to ontologies highlighting the role of stimuli, observed properties, and processes [33–35]. While each of them differs with respect to the intended application area and the definition of the core concepts, the Stimulus-Sensor-Observation ontology design pattern [36] was developed to establish a lightweight and common ground for these and future ontologies. A survey comparing the most prominent ontologies was recently presented by Compton *et al*. [37]. The major purpose for developing ontologies for the Semantic Sensor Web is to improve the discovery of sensors and observations beyond simple keyword matching or code lists. Additionally, rule-based systems can be used to discover hidden information or reclassify the data according to the user’s needs. Sheth *et al*. [28], for instance, have proposed an approach to detect Blizzard conditions based on sensor data from weather stations, a sensor and weather ontology, as well as a set of rules. McCarthy *et al*. [38] built a spatial decision support system for near-real-time hazard monitoring based on ontologies for O&M and SensorML. Sensor data is automatically converted to the ontologies for enriching the information content and allowing rule-based inference techniques to facilitate decision making.

With the advent of Linked Data [39], the mostly top-down driven research on the Semantic Sensor Web shifted towards making sensor data available following the Linked Data principles, *i.e.*, by providing Uniform Resource Identifier (URI) for raw data, dereference them via HTTP, and linked to other external data [40–45]. Today, SWE plays a major role in providing sensor related data in an interoperable way, hence, various researchers have investigated how to integrate the Semantic SensorWeb with Spatial Data Infrastructures or OGC services in general [41,45–47]. On a higher abstraction level, the term *Semantic Enablement* describes a family of models, services, ontologies, and annotations to connect the OGC driven Geospatial Web with the Semantic Web [47].

## 3. Requirements for a Sensor Plug & Play on the Sensor Web

In this section, we describe the common understanding of plug & play for devices, and for sensors in particular, by focusing on technologies such as USB and IEEE 1451. Derived from the general conception of plug & play, we define the meaning of *Sensor Plug & Play* for the Sensor Web and identify functional requirements.

### 3.1. Requirement for a Publish/Subscribe Mechanism

The common notion of plug & play describes it as the ability to make the functionality of a device instantaneously available within a system or network. In day-to-day life, plug & play is often related to devices with a Universal Serial Bus (USB) [48] connector. USB supports easy usage and configuration of external computer devices without the need for user intervention. With its support for *hot-plug* (no need for restarting the computer to use the device) and automatic configuration, USB has become the main standard for computer periphery. Once a user plugs a device into the system, the device registers itself at the standard port of the host and the configuration is initiated. According to the USB specification, the host is able to retrieve the capabilities with vendor and product identification from the device. This identification is globally unique so that operating systems can load the according driver and make the device available to the system resources via the system bus.

Our work aims at a plug & play for the Sensor Web based on OGC’s SWE framework. In analogy to USB, the SWE services represent the system resources to which the sensor needs to be made available. A new sensor should be able to automatically register with SWE services which have announced interest in its characteristics (e.g., observed phenomenon, type of sensor, or current geographic location). Thus, from a *service provider* perspective, the key requirement is:

**Requirement 1.** *A plug & play mechanism for the SensorWeb shall allow a service provider to subscribe a service on the Sensor Web by specifying sensor characteristics relevant for this service.*

From a *sensor provider* perspective, two requirements are derived:

**Requirement 2.** *A plug & play mechanism for the Sensor Web shall allow a sensor provider to publish the availability of a sensor by providing a description of the sensor characteristics.*
**Requirement 3.** *A plug & play mechanism for the Sensor Web shall allow a sensor provider to publish measured sensor data so that interested services can retrieve the data.*

Given the above requirements for sensor and service provider, the key requirement for the system architecture is:

**Requirement 4.** *A plug & play mechanism for the Sensor Web shall ensure that newly available sensors which satisfy the characteristics required by a service are automatically registered at this service and their observations are automatically inserted.*

### 3.2. Requirement for a Generic Driver Mechanism for Sensors

Once the publish/subscribe mechanism is in place, it is essential that messages between sensors and SWE services can be exchanged. A translation between heterogeneous sensor protocols and the SWE protocols is required. This translation can be generally addressed from two directions. On the one hand, it can be approached by implementing interoperable interfaces on the sensor. On the other hand, it can be approached by introducing mechanisms which bridge between the variety of sensor protocols and SWE specifications.

The first direction about interoperable interfaces is addressed by several standardization approaches. The most prominent standardized interface for sensors is IEEE 1451 [49,50]. The IEEE 1451 family of standards allows for connecting sensors to sensor networks through vendor independent interfaces. A key role within this standards family plays the Transducer Electronic Data Sheet (TEDS) which contains metadata about the sensor. TEDS are stored on small non-volatile memory devices (e.g., an EEPROM) attached to sensors. Their structure is optimized for low memory usage and only a limited set of metadata can be included, for example, sensor identification, calibration, correction data, measurement range, and manufacturer related information. Due to the focus on compactness, not all characteristics of a sensor can be captured. For example, metadata for automated processing of sensor data to higher level protocols cannot be described in TEDS. Hence, this work is based on SensorML as it has a richer data model. Hu *et al.* [51] convert TEDS to SensorML by creating a knowledge base which maps each TEDS property to an appropriate SensorML description. Extending this approach to combine it with our work to automatically generate Sensor Interface Descriptors for IEEE 1451 sensors could be promising. SID interpreter can then connect IEEE 1451 sensors on-the-fly with SWE services.

However, until now, the IEEE 1451 standards have not achieved broad acceptance. Sensor manufacturers hesitate to change their existing and well established sensor interfaces and protocols to conform to the IEEE 1451 standards. In fact, a huge variety of non-standardized sensor interfaces are in use. Different approaches try to bridge between these proprietary sensor protocols and higher level SensorWeb specifications. For example,Walter and Nash [52] analyze system designs which may lower the implementation barrier for integrating sensors and SWE services. Lightweight SWE connectors are suggested which convert raw sensor formats to SWE-based data models. The authors describe design approaches for such SWE connectors, but do not describe their implementation. Similar approaches are the AnySen application [53] and the Sensor Abstraction Layer (SAL) [54]. They introduce mechanisms which are capable of interpreting data from sensors by utilizing descriptions of the sensor. These approaches are supposed to make use of SensorML to describe sensor characteristics and thereby abstract from the interface of a sensor. However, both do not detail how the essential information, for being able to interpret incoming sensor data, is reflected within SensorML.

The systems described above act as intermediary brokers between sensors and SWE services. They take the burden of adapting the sensor protocol from the sensor manufacturer or provider. We propose to follow this idea, since standardized interfaces for sensors, such as IEEE 1451, are not yet established. A description of a sensor interface which follows a well-defined model needs to also explain how to interpret the sensor protocol. With increasing expressiveness of such a model, more complex sensor interfaces can be connected. Such an interface description has to be exchangeable in case the sensor interface is adjusted (e.g., due to firmware updates). Moreover, the interface description needs to be shareable so that, once defined, it can be passed on to others who operate sensors of the same type. Thus, we deduce the following, requirement:

**Requirement 5.** *A plug & play mechanism for the Sensor Web shall rely on a “driver” mechanism which enables the translation between a sensor protocol and the higher level SWE protocols based on a generic, exchangeable and sharable interface description associated with the sensor.*

### 3.3. Requirement for a Matchmaking Mechanism

Approaches such as USB or IEEE 1451 enable the connection of hardware devices with computer systems. However, to enable plug & play of sensors within the Sensor Web, we need to go beyond the integration of the hardware device provided through a driver mechanism (Section 3.2). In applications such as environmental monitoring, early warning, or disaster management, the integration of the sensor with the model of the specific application plays a crucial role. O&M and SOS as part of the SWE framework (Section 2) are such data / service models that serve as generic foundations for application specific models. An example for an application specific extension of O&M is WaterML 2.0 [55] for the hydrology domain.

Based on the required publish/subscribe mechanism (Section 3.1), we envision that SWE services can subscribe sensors based on their characteristics. The key challenge is to assure that these characteristics are advertised by a sensor and *match* those required by the service. For example, a sensor may characterize itself by stating its identifier, name, model number, or contact information about its provider. While a matching of this kind of *basic* characteristics can be checked by simple string comparison, *spatial*, *temporal*, and *thematic* characteristics require more advanced match techniques. Temporal characteristics include the sampling rate or the lifetime of a sensor. Spatial characteristics include the current position of a sensor, a path along which it is traveling, or a geographic region within it can function. The central thematic characteristic of a sensor is the observed phenomenon (e.g., water temperature) as its output, in combination with the unit of measure (e.g., Kelvin). But also other thematic characteristics such as the sensor configuration (e.g., the calibration), sensor deployment (e.g., distance to a road network, mobile/stationary sensor), or the measurement technique (e.g., usage of a 3D ultra-sonic anemometer or a 2D mechanical anemometer for observing wind) are important. Matching sensor characteristics with the requirements of a service is challenging and frequently requires semantic mediation; a detailed analysis has been recently described by Bröring *et al.* [8].

Sensors gather data by observing stimuli emitted from the physical environment [36]. The digital counterparts of entities in this environment are the *features of interest* residing on the Sensor Web (Section 2). Before registering a service, the service provider defines these features, e.g., for the Norwegian Sea, based on the application area. Sensors are not aware of these features as they collect field data. Hence, when subscribing new sensors, the features whose qualities are observed have to be determined. Sampling points, *i.e.*, locations at which sensors take measures, can be reduced to their spatial footprint. However, to ultimately determine the domain feature, e.g., a waterbody, its thematic component needs to be incorporated as well.

**Figure 4.**
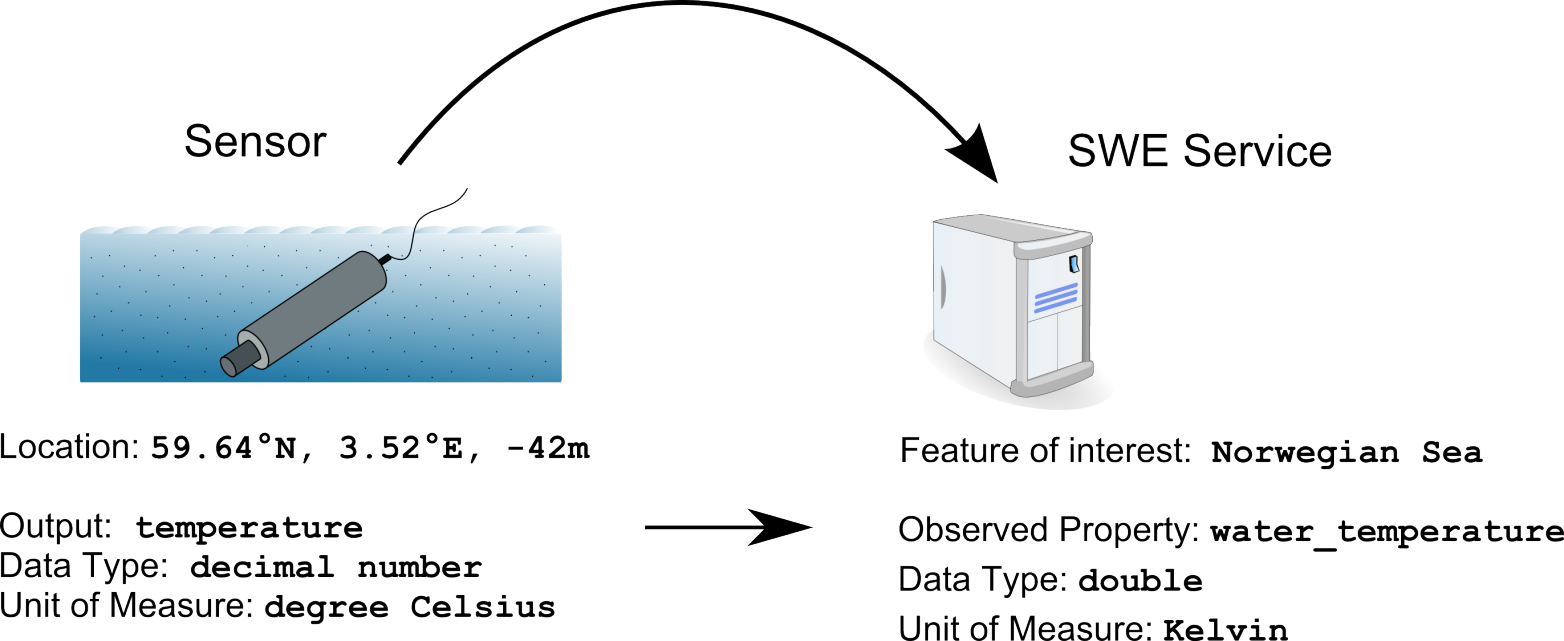
Matching between sensor and service characteristics.

Equally important is the matching of the output of a sensor and a property of a feature of interest; see Figure 4. By assuring this matching, sensor data is linked to the application model of the service. This enables the integration of the sensor with the Sensor Web. Syntactic matching is a first step in this process. Data encoding, structure, and data types of the sensor output and the feature property have to match. Second, the semantics of the output and the observed property have to be compared. Wind direction has been used to demonstrate the involved challenges. Syntactically, wind direction can be represented, for example, as a numeric value, a textual value, or even a complex data type. The semantics of wind direction can be defined as the direction *from* which the wind blows, or as the direction the wind is blowing *to* [56]. This mismatch cannot be discovered by syntactic matching alone and would lead to wrong results in later processing steps. Consequently, we deduce the following requirement:

**Requirement 6.** *A plug & play mechanism for the Sensor Web shall implement a matching between the characteristics advertised by a sensor and the characteristics required by a service.*

Semantic matching is based on the assumption that shared domain vocabularies are present and used for the semantic annotation of both the advertised and requested sensor characteristics. Semantic annotations establish links from application-specific metadata to commonly accepted vocabularies which capture the knowledge on the domain level (e.g., oceanography, hydrology, or meteorology). What vocabulary to use depends on the scenario or the required expressiveness and cannot be specified beforehand. Some vocabularies are widely accepted and can be considered as de-facto standards (some are even maintained by standardization organizations). If other, more specific, or local vocabularies are used, they need to be related to these de-facto standards. Such mappings between vocabularies ensure that reasoning engines are able to compute matching across different domains and information communities. The following requirement ensures that semantic matching can be performed:

**Requirement 7.** *A plug & play mechanism for the Sensor Web built on semantic matching shall either use a widely accepted vocabulary or more specific, but aligned vocabularies for the semantic annotation of sensor characteristics.*

## 4. A Standards-Based Architecture for Semantically-Enabled Sensor Plug & Play

In this section, we present the architecture for realizing Sensor Plug & Play on the Sensor Web. The following subsections detail how the developed components and their interplay fulfill the identified requirements and how they relate to SWE standards (Section 3).

### 4.1. Realizing a Publish/Subscribe Mechanism with the Sensor Bus

**Figure 5.**
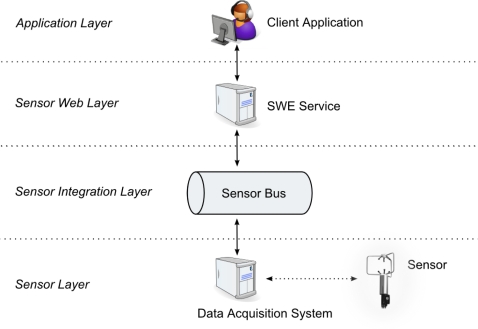
The sensor infrastructure stack.

The required publish/subscribe mechanism (Section 3.1) is realized by introducing an intermediary *sensor integration layer* between the sensor layer and the Sensor Web layer; see Figure 5. This intermediary layer is externally designed as a logical bus, the Sensor Bus. The Sensor Bus has initially been defined and implemented by [10]. In the following, an enhanced bus message protocol adjusted to the needs of semantic matchmaking and enabling Sensor Plug & Play is described. An implementation of the Sensor Bus concept based on XMPP is described in Section 5 and examples illustrating the usage of the message protocol are given in Section 6.

**Figure 6.**
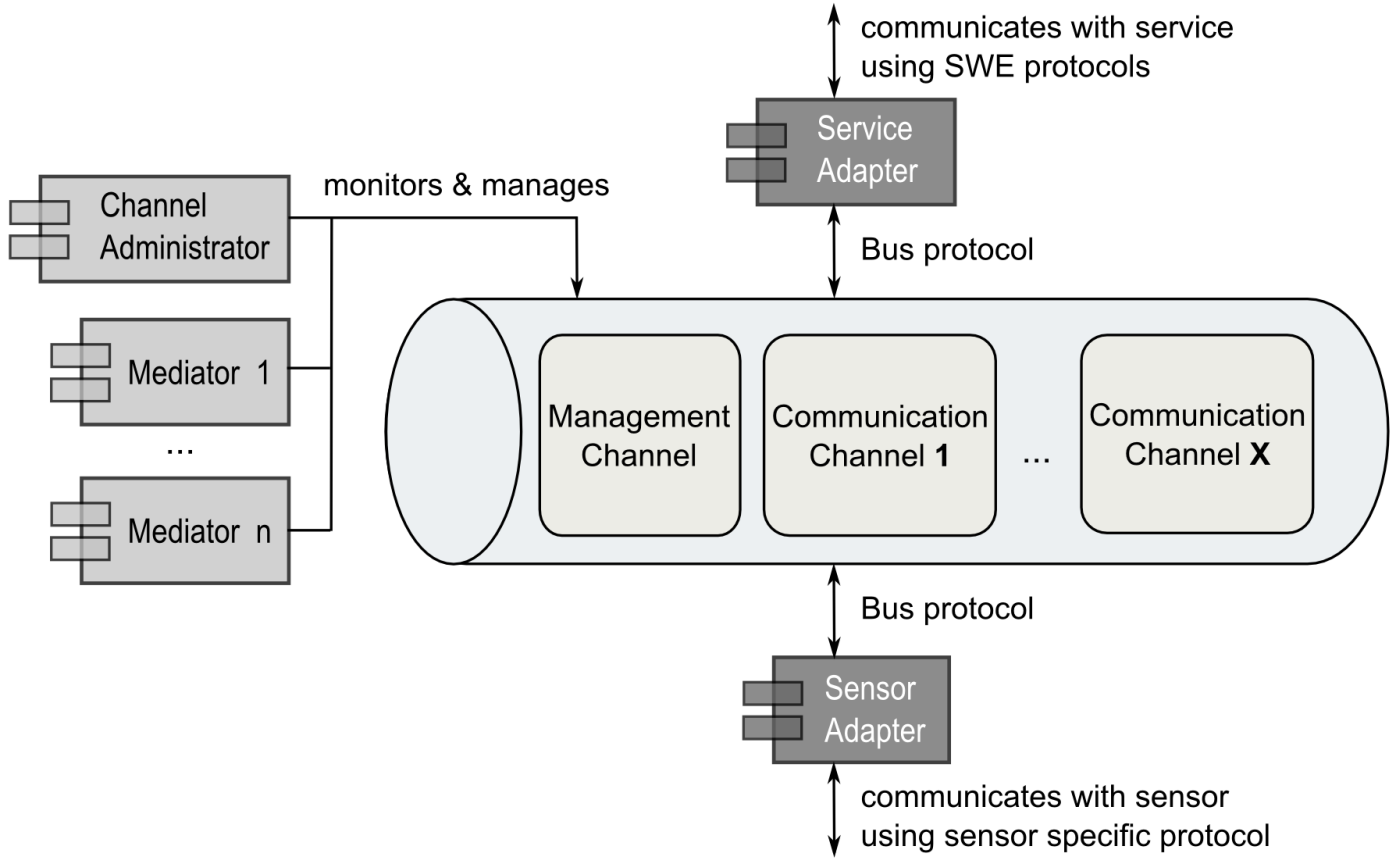
Components of the Sensor Bus.

Aligned with the *message bus pattern* [57], the Sensor Bus incorporates (1) a common communication infrastructure, (2) a shared set of adapter interfaces, and (3) a well-defined message protocol. The common communication infrastructure is realized through an underlying messaging technology. The Sensor Bus is independent of the underlying messaging technology which can therefore be exchanged. It can, for example, be realized with instant messaging systems such as XMPP [58] or IRC [59], but also using Twitter as shown in [10]. Services as well as sensors can publish messages to the bus and are able to subscribe to the bus for receiving messages in a push-based communication style. The different components (*i.e.*, sensors and Sensor Web services) can subscribe and publish to the Sensor Bus through *adapters*. Those adapters convert the service or sensor specific communication protocol to the internal bus protocol; see Figure 6).

A detailed analysis of interactions between the sensor layer and the Sensor Web layer, which emerge when introducing the Sensor Bus as an intermediary layer, is conducted by [60]. Those interactions are realized through particular bus messages which are described below. The Sensor Bus message protocol is designed in a compact style to preserve system resources. Single message fields are divided by a separator sign (the “*” character). In the following specification of the message protocol, we put variables which act as placeholders for actual values in “*<*” and “*>*” brackets.

#### **Listing 1**. The connect sensor message

C o n n e c t S e n s o r  *  <s e n s o r  d e s c r i p t i o n  URL>

To register a sensor at the Sensor Bus, the adapter belonging to the sensor sends a *ConnectSensor* message (Listing 1) which contains a URL pointing to a sensor description document. This document is placed at a Web-accessible location by the sensor publisher beforehand. The URL of the document identifies the sensor in the subsequent communication via the Sensor Bus. We propose to use the SensorML standard (Section 2) as the format of the sensor metadata document. SensorML is domain independent which makes it generic and, thus, information can be encoded in multiple ways. We demand that the sensor description conforms to a profile elaborated in previous work [61] which restricts the genericness of SensorML. By complying to this profile, all components of the architecture can rely on finding the sensor characteristics in well-defined elements of the SensorML document. These characteristics suffice for effective discovery and include [62]:

- Identification of the sensor.
- Keywords that describe the sensor.
- Contact information about the company/individual that operates the sensor.
- Capabilities of the sensor (e.g., measurement capabilities such as accuracy, or operational capabilities such as survival range).
- Classification of the sensor (e.g., model number or sensor type).
- Location of the sensor.
- Outputs of the sensor.

The connection of a service at the Sensor Bus and the subscription for certain sensor characteristics is conducted by a sequence of messages sent by the service adapter (Listing 2). First, the service adapter calls the *ConnectService* message to publish the endpoint of the service (*i.e.*, its URL) which identifies the service in future messages. Subsequently, multiple *SubcribeService* messages can be sent over the bus, each defining a subscription of the service for sensors with a particular set of characteristics.

#### **Listing 2**. The message sequence for service connection and subscription

1.  Co n n e c t S e r v i c e  *  <s e r v i c e  URL>  2.  S u b s c r i b e S e r v i c e  *  <s e r v i c e  URL>     *  <s e n s o r  c h a r a c t e r i s t i c  A>  *  <v a l u e>     *  <s e n s o r  c h a r a c t e r i s t i c  B>  *  <v a l u e>    . . .x.  S u b s c r i b e S e r v i c e  *  <s e r v i c e  URL>    *  <s e n s o r  c h a r a c t e r i s t i c  Y>  *  <v a l u e>    *  <s e n s o r  c h a r a c t e r i s t i c  Z>  *  <v a l u e>    . . .

The service subscription is specified in a key-value-pair style that associates a sensor characteristic’s unique identifier with a value. A *SubscribeService* message can contain multiple key-value pairs which are combined by a logical AND to a single subscription. This way, a service adapter can announce interest in sensors with certain survival capabilities (e.g., battery lifetime greater than 10 days) that are observing a particular *observed property* (e.g., temperature) with data being published in a particular *unit of measure* (e.g., degree Celsius).

To reference a particular characteristic in a subscription, two alternatives exist. For commonly used characteristics, such as unit of measure and observed property, we have defined a list of unique identifiers (e.g., *uom* for unit of measure). To subscribe for characteristics which are priorly unknown to the system (e.g., the survival range of a sensor) a unique concept identifier has to be used. During matchmaking (Section 4.3) such priorly unknown characteristics are compared to the generic capability elements of a sensor’s SensorML document. To make the values of these characteristics interpretable to the components listening on the bus, they are encoded as SWE Common simple types (e.g., Boolean, Text, QuantityRange) [24,63].

The message sequences described above are published in the *management channel* of the Sensor Bus, where *management components* are listening. Two kinds of management components with different responsibilities are defined; see Figure 6.

First, a *mediator* component computes the matchmaking between the characteristics a service requires and the characteristics advertised in the SensorML document of a sensor. The mediation continues if all characteristics of a sensor either match directly the requirements of a service, or a rule is available that converts between two characteristics. If the matchmaking requires a conversion rule, the service adapter needs to be notified that a transformation of incoming measurements will be required in the future. Therefore, the mediator publishes required conversion rules to the management channel. Typically, such conversion rules translate between different units of measure (e.g., Kelvin to Celsius) or different data types (e.g., decimals to categorical values). This process of semantic meditation is similar to formerly developed approaches for the semantic matchmaking of user requirements against web service capabilities, for example described by Schade *et al*. [64]. Our approach is particularly designed for the mediation between sensors and services. It is detailed in Section 4.3.

The start of the above outlined process is indicated by a *MediatingService* message (Listing 3) from the mediator to declare that it takes over the computation of the matchmaking and prevent that multiple mediators start this expensive calculation. After the process, a *Mediate* message is sent to publish the result of the matchmaking. If needed, a conversion rule is contained in the mediate message (Listing 3). The service adapter applies this rule to data received from the particular sensor to translate between its output format and the required format of the observed property on the service side. The message contains the advertised sensor output and the required observed property to which the mediation belongs. Multiple mediator instances are listening to the management channel. This way, the system is scalable and the mediation process does not cause delay. Also for the sake of scalability, the functionality of mediation and channel administration are kept in separate components.

#### **Listing 3**. The mediate message sequence

1.  M e d i a t i n g S e r v i c e  *  <s e r v i c e  URL>  2.  M e d i a t e  *  <s e n s o r  d e s c r i p t i o n  URL>  *  <s e r v i c e  URL>    *  <a d v e r t i s e d  s e n s o r  o u t p u t>    *  <r e q u i r e d  o b s e r v e d  p r o p e r t y>  [*  <c o n v e r s i o n  r u l e  >]

The second kind of management component is the *channel administrator* that monitors the management channel and is responsible for registering services and sensors to particular *communication channels*. The communication channels are used to publish and receive measured sensor data. For each output of a sensor, the channel administrator creates a new channel. By means of the *DirectSensor* (Listing 4) message, the sensor is directed to a channel where it shall publish a particular output. A service is directed to that channel if, as published in the mediate message (Listing 3), a matching between the advertised sensor characteristics and the service requirements does exist. The channel administrator directs the service by means of the *DirectService* (Listing 5) message.

#### **Listing 4**. The direct sensor message

D i r e c t S e n s o r  *  <s e n s o r  d e s c r i p t i o n  URL>  *  <a d v e r t i s e d  s e n s o r  o u t p u t>  *  <c h a n n e l>

#### **Listing 5**. The direct service message

D i r e c t S e r v i c e  *  <s e r v i c e  URL>  * <r e q u i r e d  o b s e r v e d  p r o p e r t y>  *  <c h a n n e l>

To publish new data, a sensor adapter transmits a *PublishData* message (Listing 6) via the Sensor Bus containing a time and location tag (encoded as ISO 8601 [65] and EPSG 4326), the observed phenomenon as the sensor’s output, and the data value itself. A service adapter, receiving this message, first applies the conversion rule(s) to the data and then transforms the message to the service specific protocol. In case of an SOS service adapter, the publish data message is translated to an *InsertObservation* request (see Listing 16 for an example) to upload the data to the SOS.

#### **Listing 6**. The publish data message

P u b l i s h D a t a  *  <s e n s o r  d e s c r i p t i o n  URL>  *  <t i m e  t a g>  *  <l o c a t i o n  t a g>  *  <a d v e r t i s e d  s e n s o r  o u t p u t>  *  <d a t a>

### 4.2. Realizing a Generic Driver Mechanism with Sensor Interface Descriptors

**Figure 7.**
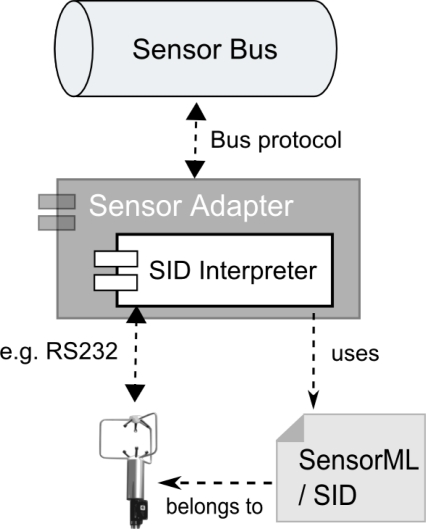
SID interpreter as a sensor adapter for the Sensor Bus.

Before a sensor can be integrated with the Sensor Web, a driver is required which understands the native sensor protocol and offers a well-defined interface that makes the functionality of the sensing device available to the outside (Section 3.2). Since there are numerous kinds of environmental sensors with various interfaces available, we propose the usage of a generic driver mechanism for sensors, as stated by Requirement 5. The Sensor Interface Descriptor (SID) model described in our previous work [11,66] can be used to provide this functionality. The SID model supports the declarative description of sensor interfaces. It is designed as a profile and extension of OGC’s SensorML standard (Section 2). An instance of the SID model, designed for a particular type of sensor, defines the precise communication protocol, accepted sensor commands, or processing steps for transforming incoming raw sensor data. Based on that information, a so-called SID interpreter is able to establish the connection to a sensor and translates between the sensor protocol and a target protocol. For this work, we have developed an SID interpreter used to build a generic sensor adapter that converts received sensor data to the Sensor Bus protocol; see Figure 7.

**Figure 8.**
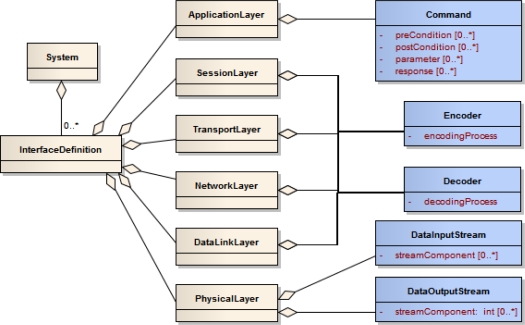
Overview of SID extension to SensorML.

SID interpreters can be built independently of particular sensor technology since they are based on the generic SID model. Figure 8 depicts an excerpt of this model. The blue colored, SID specific classes extend the beige colored classes defined in SensorML. The SID is strictly encapsulated within the *InterfaceDefinition* element of a SensorML document. Since the SID is designed for a certain type of sensor and not for a particular sensor instance, this encapsulation makes the interface description independent of the rest of the SensorML document. Consequently, it is easily exchangeable and can also be reused in SensorML documents of other sensors of the same type.

The SID model extends the elements of the Open Systems Interconnection (OSI) reference model [26] which are already contained in SensorML and associated with the interface definition. The OSI model is the basis for designing network protocols and therefore consists of a number of layers. On the lowest layer, the physical layer, the structure of the raw incoming and outgoing sensor data stream is described. This includes the definition of block identifiers and separator signs within the data stream. Next, encoding and decoding steps can be applied to the raw sensor data. Therefore, according processes can be specified and attached to the data link, network, transport, and session layer. Such processing steps are for example character escaping or checksum validation which are necessary for reliable communication with sensors. Finally, the application layer can be used to define commands accepted by a sensor, including their parameters, pre- and post-conditions, as well as response behavior. Those command definitions can for example be used by a Sensor Planning Service (Section 2) to provide an interoperable interface for tasking. A detailed description of the model can be found at [11,66].

Sensor interfaces and communication protocols are often complex. Consequently, the design and manual creation of SID instances is not straightforward. Hence, a visual SID creator has been developed [67]. This graphical tool supports users in describing the sensor interface and generate SID instances for their sensors. The creator can be used by sensor manufacturers to create SIDs for their products and provide them to clients for an easy integration of their sensors with the Sensor Web.

### 4.3. Realizing a Matchmaking Framework with Semantic Mediators

Based on the matchmaking requirements specified in Section 3.3, we introduce the semantic alignment, matching, and mediation framework for sensors and their observations.

The channel administrator links sensors and services by directing them to the same channel given that the offered sensor characteristics match the requirements of the service; see Section 4.1. This match is determined by *mediators* subscribed to the management channel. Once a new sensor is advertised by a *ConnectSensor* message, a mediator sends the *MediatingService* message and starts computing the matchmaking. Each mediator maintains a list of subscribed services and their requested characteristics. The degree of matching is computed by extracting metadata from the sensors’ advertised SensorML document and validating them against the characteristics requested by the services via *SubscribeService* messages (Section 4.1).

The mediators can combine different levels of matchmaking ranging from syntactic, keyword-based matching to semantic alignment and meditation using Semantic Web ontologies and technologies. In fact, most existing solutions combine a multitude of approaches [68–74]. On the lowest level, a syntactic metric such as the Levenshtein distance can be used for the string-based comparison of the sensor’s advertisement *adv* and service’s requirements *req*. The previously specified Requirement 6, however, calls for extended temporal, spatial, and semantic matching and mediation functionalities. In this work, we focus on the semantic component; realizing the temporal and spatial matchmaking follows similar mechanisms based on qualitative or quantitative reasoning. For example, this kind of matching can be used to determine whether the region covered by a specific sensor is spatially contained within the area represented by a feature of interest associated with a service. In a quantitative case, features can be directly compared based on their geometries; in a qualitative setting, a region connection calculus, e.g., RCC8 [75], can be used.

Semantic matchmaking requires the advertised and requested characteristics to be expressed in a formal language supporting inference. The mediator performs two steps, *concept creation* and *semantic matchmaking*, before it publishes the matching response on the Sensor Bus. The first step includes (1) the creation of the *req* sensor template (including a set of concepts and individuals) from the characteristics announced by the service adapter in the *SubscribeService* message (Listing 2) and (2) the creation of *adv* sensor template from sensor characteristics extracted from the SensorML document. The created ontology elements are aligned with the OWL-based ontology developed by the W3C SSN-XG [32,36,76], which implements the Sensor-Stimulus-Observation (SSO) ontology design pattern; see Section 2.2. Those ontologies provide a rich set of concepts and relations and are used to model measurement properties such as latency, operating properties (e.g., power range), survival properties (e.g., battery lifetime), and many more.

The semantic matchmaking results can be further improved if the requested and advertised characteristics are semantically annotated, *i.e.*, extended with references to concepts from shared domain vocabularies. The SSN ontology does not specify types of observed properties but introduce the generic *Property* concept for further subclassing. Properties and feature types can be imported from other ontologies, e.g., SWEET [77] for environmental terminology or the vocabularies provided by the Marine Metadata Interoperability Project [78].The concept creation takes semantic annotations into account by aligning the created concepts with the annotated concepts. The Alignment API and server can be used to integrate multiple ontologies if no predefined mappings exist, see [73] for details. With semantic annotations in place, advertised characteristics such as the sensor’s observed property are used to compute a match with the service requirements template.

In the matchmaking step, the mediator reclassifies [64,79] the service ontology by injecting the *adv* sensor concept. The service ontology may already store a list of previously requested sensor concepts. The reclassification is based on subsumption reasoning and can result in either of the following matches (see also [80] for a more detailed description of matching types). A detailed example is given in Section 6 and illustrated in Figure 12:

**exact match:** This occurs if the sensor concept *adv*, defining the advertised sensor characteristics, is equivalent to the concept of the service’s sensor template *req*, defining the required sensor characteristics, *i.e.*, if *adv* ≡ *req*.
**plugIn match:** In this case, *adv* has been reclassified as subconcept of *req*, *i.e.*, *adv* ⊑ *req*. Consequently, the characteristics requested by the service are less specific. For example, requested observed property is *Temperature*, while the advertised sensor output is a more specific kind of temperature (e.g., water temperature). Since *WaterTemperature* is modeled to be a subconcept of *Temperature*, the reasoner infers a successful match.
**subsumed match:** In this case, *adv* has been reclassified as superconcept of *req*, *i.e.*, *adv* ⊒ *req*. Therefore, *req* is more specific than *adv*. For example, a sensor measures temperature with a sampling rate of 1 measurement per day, whereas the service requires a sampling rate of at least 1 measurement per hour. Hence, the advertised sensor properties do not meet the requirements of the service and the result is no match.
**fail:** If none of the cases above can be computed, the result of the matchmaking is negative. More specifically, no alignment between different property ontologies could be computed or *adv* and *req* are disjoint classes, *i.e.*, *adv* ⊓ *req* ⊑⊥. For example, a service requests the observed property *Temperature* but the advertised output is *WindSpeed*.

Even if the subsumption reasoning resulted in no match, the advertised and required characteristics might still match if the sensor’s output can be transformed into the requested format. For example, all described properties match, but the sensors output is expressed with a unit of measurement incompatible with the request. If the sensor announces temperature observations measured in degree Celsius, while the service expects Kelvin, a conversion would add 273.15 to the observed value and forward it to the service. Rules (e.g., Semantic Web Rule Language, SWRL) can attach these conversion methods to the individuals resulting from the concept creation phase. The executable conversion instructions are stored as literals in the ontology. This includes simple arithmetic functions such as the described unit conversion but also more sophisticated conversions, for example data type transformations, can be supported by this approach. Coming back to the wind direction example, such a conversion could transform from measured numeric values (wind direction measured in degree) to required nominal values (wind direction as North, Northeast, *etc*.).

The rules are automatically triggered during the reclassification phase. In case of a mismatch, the mediator checks if conversion instructions have been attached to the requested sensor templates. Existing conversions result in a match, and the literal containing the conversion instruction is published in the mediator’s response. An example for a mediate message to announce such a rule is given in Listing 13. Simple conversions are directly executed on runtime by the service adapter, and are encoded in MathML [81] with placeholders for the observation value. More complex conversions can be implemented by remote Web services, for example OGC’s Web Processing Services (WPS) [82]. In the latter case, the literal contains information how to invoke the service operation. Our implementation focuses on simple conversions so far. This de-coupling of the semantic matchmaking performed by the mediators and the conversion execution performed by the service adapters makes the proposed architecture for Sensor Plug & Play more robust and scalable, as the processing steps may be time consuming and their outcomes are delivered asynchronously.

In this work, we propose a subsumption reasoning-based matching that reclassifies the service provider’s ontology after injecting the *adv* sensor concept. However, this design decision does not exclude the use of other methodologies, e.g., semantic similarity measurement or probabilistic approaches, in the future. Both can improve the mediation framework by offering ranking information in addition to the boolean matching used so far. Nevertheless, from an integration point of view, subsumption-based matching is most universal as it can be applied to any combination of Semantic Web ontologies (as long at they can be aligned) and any OWL language profile. Finally, all major reasoners offer highly-optimized taxonomic reclassification as basic service via a DIG or OWLlink API [83].

## 5. Implementation

This section describes the implementation of the plug & play architecture described in Section 4. All implemented components are available as open and free source code by the 52°North Sensor Web [84] and Semantics [85] communities—the links to the software project websites are stated below.

**Figure 9.**
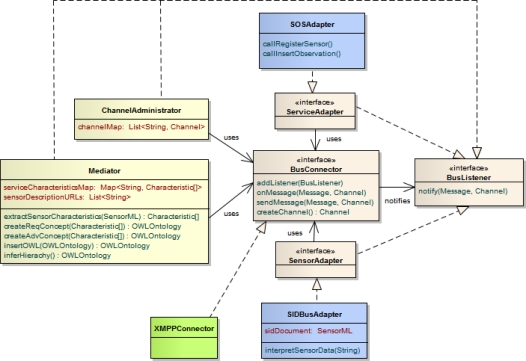
Overview of the class hierarchy of the Sensor Bus implementation.

At the core of the architecture, the Sensor Bus enables the interplay of the developed components. This is similar to an Enterprise Service Bus [86] which can be used in pure Service Oriented Architectures to establish a loose coupling between web services. We implemented the Sensor Bus [87] based on a generic structure of interfaces wrapping the communication between the involved components and the message protocol (Figure 9). The BusConnector interface provides functionality for both sending and receiving messages by applying the observer pattern [88]. An implementation of the bus connector calls onMessage() in case of an incoming message. The message is then decoded and passed on to observers. Concrete sensor and service adapters act as such observers by implementing the BusListener interface and registering them at the bus connector by means of the addListener() method. They receive incoming messages through their implementation of the notify() method, and react on it according to their specifications. The adapters transmit messages to the bus by using the sendMessage() method of the bus connector. By providing an according implementation of the bus connector interface, the Sensor Bus can be adapted to different communication infrastructures. In this work, we realized the bus connector for the Extensible Messaging and Presence Protocol (XMPP) by providing the XMPPConnector class. This way, the Sensor Bus can be set up based on an XMPP server for instant messaging.

**Figure 10.**
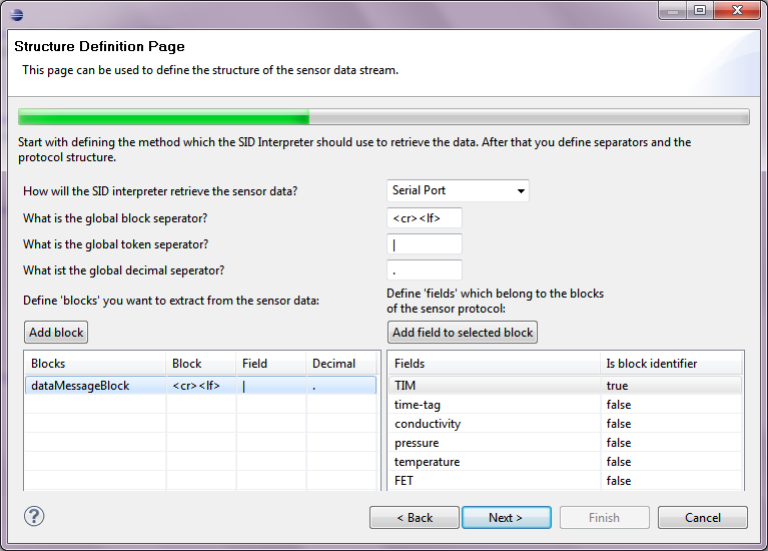
Sensor data message protocol defined in SID Creator.

The SensorAdapter interface encapsulates the connection to a sensor. In general, a sensor adapter is implemented for a certain sensor type and thus capable of translating between the sensor’s protocol and the bus protocol. In this work, however, we provide a generic sensor adapter, the SIDBusAdapter which reuses our SID interpreter implementation [89] developed in previous work [11]. By utilizing the SID interpreter, the generic senor adapter can perform the protocol translation based on an SID document (Section 4.2) belonging to the sensor. Such an SID document either already exists for the sensor and can thus be reused, or it needs to be created. In the latter case, the sensor provider can make use of our SID creator tool [67]. Figure 10 shows an excerpt of the SID creator while designing a sensor’s SID document.

The ServiceAdapter interface represents the connection of a service to the Sensor Bus. We have implemented such service adapters for the Sensor Event Service and the Sensor Observation Service (Section 2). Here, we focus on the latter one, the SOSAdapter, which calls the SOS operations RegisterSensor and InsertObservation (see Listing 16 for an example) of an SOS server in reply to a received *PublishData* message (Listing 6). The implementation of the SOS adapter utilizes the OX-Framework [90,91] as an encoding engine for the SOS operation requests and O&M observations.

Also, the classes ChannelAdministrator and Mediator as representations of the respective management components act as observers on the bus. Thus, they are notified when sensors or services connect to the bus. The channel administrator directs the registering components to channels and therefore maintains a map of sensor outputs to bus channels. If necessary it sets up new channels by calling the createChannel() method of the bus connector.

The mediator maintains a mapping of services to their required characteristics and a list of links to SensorML documents of the registered sensors, as the document URLs act as identifiers of the sensors. Further, the mediator comprises methods for the extraction of sensor characteristics from SensorML as well as the concept creation (createReqConcept() and createAdvConcept()). Also, the functionality of injecting the advertised sensor concept into a service’s sensor template and reclassifying the concept hierarchy are encapsulated in their own methods, insertOWL() as well as inferHierarchy(). For the implementation of concept creation, concept injection, and reasoning, we rely on the OWL API [92] as well as the OWL reasoner Pellet [93].

## 6. Application

This section applies our semantically-enabled Sensor Plug & Play approach to the use case of a marine oil spill in the Norwegian Sea, as introduced in Section 1. A local Sensor Web infrastructure is already in place and built upon the Sensor Bus. New sensors need to be connected and made available in an on-the-fly manner. As a proof-of-concept, we demonstrate in the following the plug & play of two CTD sensors, an *RBR XR-420* and a *Seabird SBE 37* (see Figure 1), with a Sensor Observation Service.

To connect the two CTD sensors to the Sensor Bus we utilize the generic sensor adapter which incorporates an SID interpreter (Section 4.2). The SIDs for the two sensors have been designed using the SID creator tool [67]. This tool follows the *wizard* user interface pattern. Figure 10 shows the page of the wizard which allows to define the structure of the data stream coming from the sensor. Here, the message format of the RBR XR-420 sensor for delivering measured data is defined (Listing 7). This is done by stating separator signs and associating labels to the six token fields of the message for the identification of data chunks and further processing. With this information defined and captured in an SID document the sensor adapter is capable of translating the sensor messages to the bus protocol.

### 

#### **Listing 7**. Example of a data message coming from an RBR XR420 sensor and containing values for time, conductivity, pressure and temperature

T I M  |  1 1 0 3 0 8 1 3 5 4 2 3  |  5 5 . 7 3 2  |  3 . 0 4 3  |  1 . 5 4 2  |  FET<cr><l f>

Once the sensor adapter is started and receives data from the sensor, it sends the message ConnectSensor * http://myserver.org/sensor/s1.xml to register the sensor at the bus. The SensorML document [94] to which this message points contains the metadata of the sensor. In this case study, we focus on a selected output of the sensor, namely temperature, and the sensor’s survival range (Listing 8).

The sensor output states its data type (a *Quantity*, which represents decimal numbers), the unit of measure (a UCUM [95] code, here, *Cel* for degree Celsius), and references a phenomenon. The phenomenon reference can be resolved since it links to the SWEET ontology [77] to retrieve a description.

The survival range, *i.e.*, the sea water depth up to which the sensor can be exposed to without damage, is stored as a *QuantityRange* in the generic capabilities element of the SensorML document (Section 2). Thereby, the semantics of the quantity range are declared by pointing to the concept *SurvivalRange* of the W3C SSN-XG ontology. In this example, the sensor states that it can survive up to 100 meters below sea level. Note that our framework does not enforce which ontologies should be used, e.g., for the sensor output description. The choice of an appropriate ontology depends on the use case and is up to the provider.

#### **Listing 8**. Excerpt of the used SensorML document

<s m l : **c a p a b i l i t i e s** >  <s w e : S i m p l e D a t a R e c o r d>    <s w e : f i e l d >      <s w e : **Q u a n t i t y R a n g e**  d e f i n i t i o n =” http://purl.oclc.org/net/ssnx/ssn#SurvivalRange”>        <s w e : **u o m**  c o d e =”**m**” />        <s w e : **v a l u e**>–100  0</s w e : **v a l u e**>      </s w e : **Q u a n t i t y R a n g e**>    </s w e : f i e l d >  </s w e : S i m p l e D a t a R e c o r d></ sml : **c a p a b i l i t i e s** > . . . <sml : **o u t p u t** n a m e=” t e m p”>  <s w e : **Q u a n t i t y**  d e f i n i t i o n =” http://sweet.jpl.nasa.gov/1.1/property.owl#Temperature”>    <s w e :**u o m**  c o d e =”**C e l** ” />  </s w e : **Q u a n t i t y**></ s m l : **o u t p u t**>

In reply to the connect sensor message, the channel administrator directs the sensor adapter to publish the sensor data for each phenomenon in a separate channel. For the phenomenon temperature, the according message is (Listing 9):

#### **Listing 9**. Example of a direct sensor message

**D i r e c t S e n s o r**  *  http://myserver.org/sensor/s1.xml  *  http://sweet.jpl.nasa.gov/1.1/property.owl#Temperature  *  **channe l_1**

**Figure 11.**
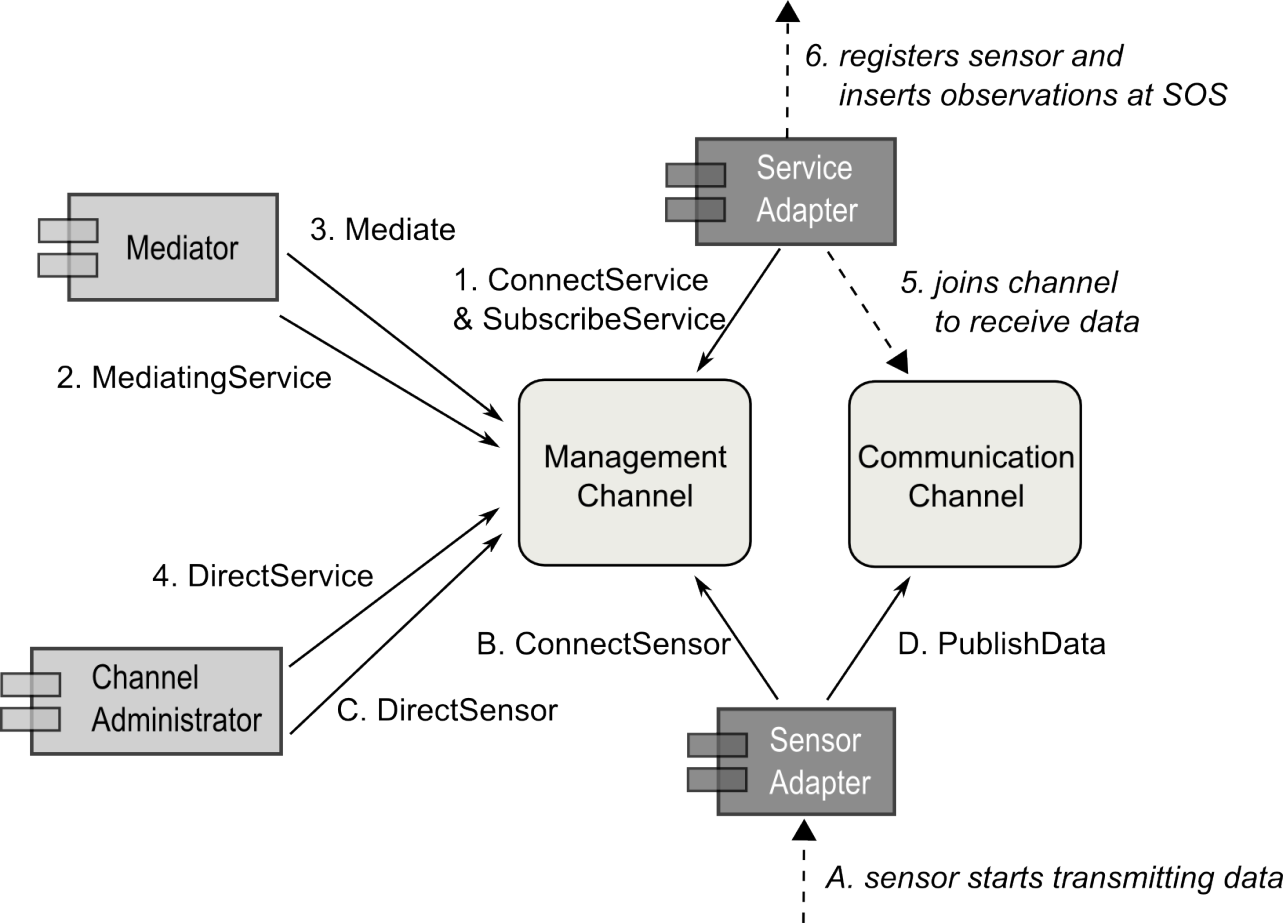
Overview of message sequences for connecting, mediating and directing sensor and service.

To give an overview, Figure 11 shows the above described message sequence for registering a sensor at the Sensor Bus (Sequence A–D). Also, the figure illustrates the sequence of messages to connect, mediate and direct an SOS (Sequence 1–6) which are described in the following. First, a service adapter registers an SOS at the Sensor Bus by, for example, subscribing it for all temperature related observations. To do so, the concept *TemperatureRelatedQuantity* is chosen, which is a super type of *Temperature* within the SWEET ontology. Further, the SOS restricts the values to Kelvin as unit and requires sensors with a survival range of at least −50 m. Therefore, the following message is sent (Listing 10):

#### **Listing 10**. Example of a subscribe service message

**S u b s c r i b e S e r v i c e**  *  http://mySOS.org  * **o b s e r v e d P r o p e r t y**  *  http://sweet.jpl.nasa.gov/1.1/property.owl#TemperatureRelatedQuantity  * **uom**  *  **K**  *  http://purl.oclc.org/net/ssnx/ssn#SurvivalRange  *  <**Q u a n t i t y R a n g e**><**uom**  c o d e = ’**m**’/><v a l u e>–50 0</ v a l u e ></**Q u a n t i t y R a n g e**>

Next, a free mediator starts computing the matchmaking between the characteristics advertised by the sensor and the characteristics required by the service (Section 4.3) and sends the mediate service message (Listing 11) to inform that it started to process the request. The template representing the sensor characteristics required by the service has been created before, and is already in the ontology. The sensor template created during the concept creation phase includes the following concepts and individuals:

**AdvertisedObservation:** The observation is the root concept of the sensor template. It is observed by the *AdvertisedSensor*, and produces an *AdvertisedSensorOutput*.
**AdvertisedSensor:** This concept represents the sensing device, which observes a phenomenon such as temperature or salinity. The sensing device has characteristics such as survival range or battery lifetime.
**AdvertisedSensorOutput:** The output of the sensing device links to the observation value.
**AdvertisedSensorOutputValue:** The output’s value is an individual parametrized by units of measure, and can also have other parameters e.g., describing the data quality.

#### **Listing 11**. Example of a mediate service message

**M e d i a t i n g S e r v i c e**  *  http://mySOS.org

**Figure 12.**
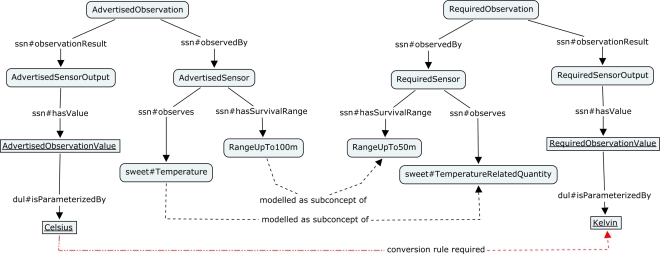
Created concepts for the *req* sensor and the *adv* sensor.

Figure 12 illustrates the different ontology elements constructed by the mediator during ontology creation phase. The *AdvertisedSensor* has the property *observes* pointing to the concept *Temperature*, while the *RequiredSensor* is associated with the *TemperatureRelatedQuantity*–both are from the SWEET ontology. Also, the two different units of measure have been included. Further, the survival range is defined in the advertised document. Hence, during the concept creation the property *hasSurvivalRange* is added to the concept *AdvertisedSensor*. This property links to the concept *RangeUpTo100m*, which is a subconcept of *RangeUpTo50m* (coming from the required sensor template). This example relies on certain ’knowledge’ of the mediator about how to transform the utilized SWE Common basic types into according concepts. Here, the mediator knows that it has to transform the data type *QuantityRange* to the Range* concepts.

The concepts are created via the OWL API [92]. The API is coupled with Pellet [93], an open-source Java OWL-DL reasoner [96], which supports both, subsumption reasoning and the execution of SWRL [97] rules [98].

After the concept creation, the matching is determined. The observed phenomenon *Temperature* has been defined for the advertising sensor. Since the SOS is requesting sensors which observe the more general property *TemperatureRelatedQuantity*, a *plugIn match* is computed, no conversion of result values is required.

**Figure 13.**
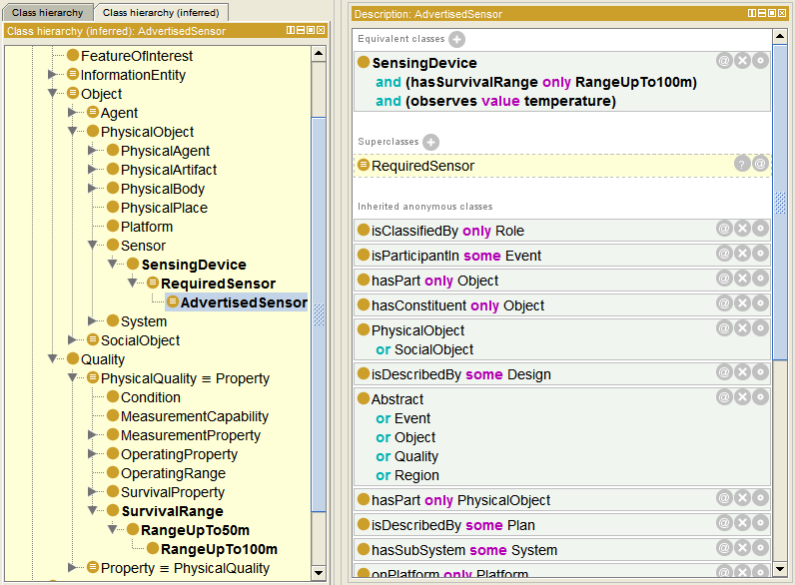
Example of a plugIn match of the survival range as screenshot in Protégé.

Taking the requested survival range into account, the service asks for sensors which can work up to at least 50 m, whereas the advertised sensor survives up to 100 meters below sea level. In Figure 13, a screenshot of the Protégé [99] ontology editor shows the results of the reclassification performed by the reasoner. Since *RangeUpTo100m* is modeled as subconcept of *RangeUpTo50m*, the advertised sensor is reclassified as subconcept of the required sensor, *i.e.*, a *plugIn match* is the result.

Finally, the matching of the unit of measures is computed. This case relies on the SWRL rules attaching the conversion instructions to the result values. The individual *AdvertisedSensorOutput* is modelled to be measured in degree Celsius, whereas the requesting service asks for Kelvin. The following SWRL rule is an example how a match can still be inferred (Listing 12):

#### **Listing 12**. SWRL rule attaching a conversion rule

O b s e r v a t i o n V a l u e ( ? x ),  i s P a r a m e t r i z e d B y ( ? x,  d e g r e e–Ce l s i u s ) –>a p p l y Co n v e r s i o n R u l e ( ? x,  D e g r e e C e l c i u s 2 K e l v i n )

Without the SWRL rule, the reasoning would result in no match due to the conflicting units of measure. However, applying the SWRL rule adds a new property *applyConversionRule* to the individual *AdvertisedObservationValue*. This new property points to the conversion instructions which have to be applied before the result values can be processed by the requesting SOS. The mediator extracts this conversion rule, and includes it in the *Mediate* message that contains references to sensor output and required observed property to which the formula needs to be applied (Listing 13).

#### **Listing 13**. Example of a mediate message with conversion rule

**M e d i a t e**  *  http://myserver.org/sensor/s1.xml  *  http://mySOS.org  *  http://sweet.jpl.nasa.gov/1.1/property.owl#Temperature  *  http://sweet.jpl.nasa.gov/1.1/property.owl#TemperatureRelatedQuantity  *  <math><mrow><mi>VAL</mi><mo>+</mo><mi>273,15</mi></mrow></math>

In response to the mediate message, the channel administrator performs a look up to which channel sensor *s1* has been directed for publishing temperature data. Consequently, the channel administrator instructs the service to join that particular channel to retrieve measurements of the *TemperatureRelatedQuantity* for which it subscribed (Listing 14).

#### **Listing 14**. Example of a direct service message

**D i r e c t S e r v i c e**  *  http://mySOS.org  *  http://sweet.jpl.nasa.gov/1.1/property.owl#TemperatureRelatedQuantity  *  **channe l_1**

For registering the sensor at the SOS, the service adapter calls the *RegisterSensor* operation which carries the SensorML document of the sensor. Subsequently, the service adapter inserts received data as observations into the SOS. An example for a publish data message sent by a sensor adapter is shown in Listing 15. The service adapter receives this message and transforms the contained temperature value to Kelvin by executing the conversion rule posted by the mediator. Finally, the service adapter transforms the received message to an *InsertObservation* request as shown in Listing 16 and sends it to the SOS. Thereby, the feature of interest is set as a sampling point with the coordinates received from the sensor.

#### **Listing 15**. Example of a publish data message

**P u b l i s h D a t a**  *  http://myserver.org/sensor/s1.xml  *  2 0 1 1–0 3–0 8 T 1 3 : 5 4 : 2 3  *  5 9 . 6 4 3 . 5 2  *  http://sweet.jpl.nasa.gov/1.1/property.owl#Temperature  *  1 . 5 4 2

#### **Listing 16**. Example of a simplified SOS InsertObservation request generated from a *PublishData* message

<s o s : **I n s e r t O b s e r v a t i o n**  s e r v i c e = ’S O S’ v e r s i o n = ’1 . 0 . 0 ’>  . . .  <**O b s e r v a t i o n**>    <**s a m p l i n g T i m e**>      <g m l : t i m e P o s i t i o n>        2 0 1 1–0 3–0 8 T 1 3 : 5 4 : 2 3      </g m l : t i m e P o s i t i o n>    </ **s a m p l i n g T i m e**>    <**p r o c e d u r e** x l i n k : h r e f =” http://myserver.org/sensor/s1.xml”/>    <**o b s e r v e d P r o p e r t y**      x l i n k : h r e f =” http://sweet.jpl.nasa.gov/1.1/property.owl#TemperatureRelatedQuantity”/>    <**f e a t u r e O f I n t e r e s t**>      <s a : **S a m p l i n g P o i n t** g m l : i d =” p1”>        <s a : s a m p l e d F e a t u r e x l i n k : h r e f =””/>        <s a : p o s i t i o n>          <g m l : P o i n t>            <g m l : p o s s r s N a m e =” u r n : o g c : d e f : c r s :EPSG: 4 3 2 6”>5 9 . 6 4 3 . 5 2</ gml : pos>          </g m l : P o i n t>        </ s a : p o s i t i o n>      </ s a : **S a m p l i n g P o i n t**>    </ **f e a t u r e O f I n t e r e s t**>    <**r e s u l t** x s i : t y p e =” g m l : M e a s u r e T y p e ” uom=”**K**”>      2 7 4 . 6 9 2    </ **r e s u l t**>  </**O b s e r v a t i o n**></ s o s : **I n s e r t O b s e r v a t i o n**>

Henceforth, the data is stored by the SOS and available to clients via its standardized interface. It can be accessed and retrieved in a pull-based manner. In a similar way, a Sensor Alert Service or Sensor Event Service (Section 2) can be registered at the Sensor Bus to provide data in a push-based manner. Those push-based services receive the incoming data, filter it by certain predefined criteria and forward it to interested clients.

## 7. Conclusion and Outlook

In this work, we identified the need for methods which facilitate the on-the-fly integration of sensors with the Sensor Web while minimizing the administration efforts. This functionality is important for applications such as disaster management where an easy integration of new sensors is required. For achieving this aim, we conducted a detailed requirements analysis. Three groups of requirements emerged, which declare the need for (1) publish/subscribe functionality for instantaneous communication between sensors and services, (2) a driver mechanism for interpreting raw sensor data, and (3) matchmaking functionality between sensors and domain specific service models. To fulfill these requirements we designed a standards- and service-based architecture for *Sensor Plug & Play*.

For realizing Requirements 1 to 5, the architecture comprises results from our previous work, the Sensor Bus combined with the interpreter for Sensor Interface Descriptors (SIDs). After bringing these technologies together and enhancing the message protocol of the Sensor Bus for enabling Sensor Plug & Play, we designed a matchmaking and mediation framework based on Semantic Web methods to fulfill Requirement 6. This framework is based on mediators which listen as encapsulated and loosely-coupled components on the bus. They perform the mediation between advertised sensor characteristics (e.g., output definition and unit of measure) and service requirements by performing concept creation and reasoning. For specifying concepts, we make use of the W3C SSN-XG ontology as well as the SWEET ontologies to represent environmental phenomena (conforming to Requirement 7). Other third-party ontologies can be integrated as well as long as they are alignable with the SSO pattern. For mediating between non-matching concepts, we added SWRL rules which define conversions, such as simple unit or data type transformation. The implemented concepts have been made available as open source software by the 52° North Sensor Web and Semantics communities.

Finally, we demonstrated the applicability of the developed approach by utilizing the implemented architecture to automatically plug two marine CTD sensors into a Sensor Observation Service. Such sensors are commonly used in oceanographic research and can for example be applied to monitor oil weathering after spills. This demonstration has shown that the proposed approach can be used to facilitate the realization of ocean observing systems [100]. A next step is to evolve our approach and apply it to further use cases (e.g., flood management).

The necessary steps for providers of services or sensors to make their components available on the Sensor Web with the presented approach can be summarized as follows. To plug in a sensor, the *sensor provider* needs to define the sensor metadata, its advertised characteristics, in SensorML following the profile for sensor discovery [61]. Additional metadata, not covered by the profile, can be described with the *capabilities* elements in the SensorML document. To make use of the generic driver mechanism, the SensorML document needs to contain a description of the sensor protocol conforming to the SID specification [66]. Then, the sensor adapter is able to communicate with the sensor based on its SID and publishes the sensor metadata by pointing to the SensorML document when sending the *ConnectSensor* message to the bus. To register a service, the *service provider* needs to specify the characteristics of sensors which the service requires. The service adapter will state these required characteristics when sending the *SubscribeService* message via the bus. The identifiers of those characteristics are either pre-defined (e.g., for unit of measure and observed property) or are generic and hence compared to the *capabilities* elements of the SensorML documents (see Sections 4.1 and 6).

Following the above steps, our approach aims at achieving plug & play for single sensors with the Sensor Web. Although, those sensors can be part of a network, the network topology is not considered. Each sensor is registered individually at the system. In future, our approach might be extended to also support plug and play of entire networks of sensors.

Of course, this article primarily describes the theoretical concepts of the developed approach for Sensor Plug & Play. In future, when this approach shall be applied in practice or commercial environments, tools and graphical user interfaces need to be present which support service as well as sensor providers in defining required/advertised characteristics of their components. This is important to minimize also the administration efforts in creating service/sensor descriptions. The developed architecture provides the framework for the development of such tools. A tool that supports a sensor provider in generating the protocol description of a sensor has already been described in the article, the SID Creator (Section 6). This SID Creator can be extended in future to also facilitate the semantic annotation of the sensor’s SensorML file.

To compute the matching between concepts from differing ontologies, we rely on semantic matchmaking computed by reasoning engines. So far we distinguish between 4 different (mis-)matches, however only exact and plugIn match allow for direct integration. Our approach may be extended by non-symmetric semantic similarity measurement to reason about the degree of matching [71]. In previous work, we have demonstrated that the SIM-DL similarity server can be used to compare classes to determine their conceptual overlap as fit-for-purpose estimator [101]. The same approach can be taken by the mediator to quantify the degree by which the sensor is a subclass or superclass of the service template, respectively. As illustrated in Section 6, the concept creation can also be applied to quantitative properties such as the sensor’s survival range if it follows nested classes approach [102]. This might potentially result in large ontologies (since each numeric value results in a new concept), which can have an impact on the reasoning performance. The performed mediation during the subscription and transformation during the publication of new observations are not communicated to the end-user. This might be a problem for complex transformations which, for example, aggregate information from different sources. Here, the resulting observation data might be extended with data quality parameters explaining the performed transformation steps. For simple transformation, as the mentioned conversion in between measurement units, the rules can be embedded as MathML statements in the domain ontologies. Creating these rules remains the responsibility of the authorities maintaining the domain ontologies. In future, this burden may be relieved by processes that, for example, automatically integrate unit conversion rules from public GML unit dictionaries [103] into the ontologies.

In coming applications, the presented semantic matching and mediation approach can be put to use in on-stream processing. This may include the dynamic fusion of incoming data streams to aggregated observations, for example the combination of temperature and conductivity data streams measured by a CTD underwater sensor to derive a stream of salinity measurements. Similar to earlier approaches (e.g., [53]) where sensor fusion has been done on the web service level, this approach performs the fusion before the data is ingested by the service. To realize such functionality, the simple MathML encoded conversion rules, which are determined in our current implementation, need to be replaced by a more powerful transformation language.

A further topic for future developments is to incorporate spatial and temporal reasoning functionality. In the requirements analysis for a matchmaking mechanism (Section 3.3), we have identified the need to determine the domain feature which a sensor observes. Although our current implementation does not support such mediation yet, the proposed matchmaking framework is flexible enough to incorporate such functionality.

Additionally, mechanisms to assign trust levels to sensors and sensor providers need to be elaborated. Trust may depend on the quality of observations of previously plugged in sensors. Determining the level of trust requires validation algorithms that take into account observations from calibrated sensors but also trust and metrics for deciding whether observations are suitable for the validation process [104].

Scalability is addressed by the architecture design through de-coupling and encapsulating the tasks of match computation and rule execution. While the first is performed by mediators, the latter is in responsibility of the service adapters. Mediators as well as service adapters can run on separate machines. If increasing numbers of sensors register at the Sensor Bus, additional mediators can be mounted to the bus to avoid latency in match computation. As soon as a mediation needs to be processed, an idle mediator reacts and declares its responsibility by sending the *MediatingService* message. Since potentially unlimited mediators can be mounted to the bus, the architecture scales. Also, the designed message protocol is kept light-weight to save system resources (e.g., on the sensor side). Due to this simplicity of the protocol, not only the here used XMPP but also other underlying messaging platforms are possible.

Alternatively, a future development could built the designed architecture on OGC’s Sensor Event Service (SES) [20]. The SES realizes a publish/subscribe architecture based on Web Service Notification [105]. A benefit would be to gain from the SES’s rich filter functionality which enables Complex Event Processing (CEP) [106] on incoming sensor data. However, since the SES is associated with a single event processing engine, such an approach would be contrary to the scalability achieved from the de-coupled mediators.
